# Plumbagin is a NF-κB-inducing kinase inhibitor with dual anabolic and antiresorptive effects that prevents menopausal-related osteoporosis in mice

**DOI:** 10.1016/j.jbc.2022.101767

**Published:** 2022-02-27

**Authors:** Gengyang Shen, Xin Liu, Wei Lei, Rong Duan, Zhenqiang Yao

**Affiliations:** Department of Pathology and Laboratory Medicine, and Center for Musculoskeletal Research, University of Rochester Medical Center, Rochester, New York, USA

**Keywords:** osteoporosis, NF-κB-inducing kinase (NIK), osteoclast, osteoblast, small molecular compound, ALP, Alkaline phosphatase, BFR, Bone formation rate, BdMPCs, Bone-derived mesenchymal progenitor cells, BM, Bone marrow, BPs, Bisphosphonates, BV/TV, Bone volume/tissue volume, M-CSF, Macrophage colony-stimulating factor, MAR, Mineral apposition rate, MSC, Mesenchymal stem cell, NIK, NF-κB-inducing kinase, OB, Osteoblast, OC, Osteoclast, OVX, Ovariectomy, RANKL, Receptor activator of nuclear factor kappa-B ligand, Tb.N, Trabecular number, Tb.Th, Trabecular thickness, TRAP, Tartrate-resistant acid phosphatase

## Abstract

Osteoporosis is caused by enhanced bone resorption and relatively reduced bone formation. There is an unmet need to develop new agents with both antiresorptive and anabolic effects to treat osteoporosis, although drugs with either effect alone are available. A small molecular compound, plumbagin, was reported to inhibit receptor activator of nuclear factor kappa-B ligand–induced osteoclast (OC) differentiation by inhibiting IκBα phosphorylation–mediated canonical NF-κB activation. However, the key transcriptional factor RelA/p65 in canonical NF-κB pathway functions to promote OC precursor survival but not terminal OC differentiation. Here, we found that plumbagin inhibited the activity of NF-κB inducing kinase, the key molecule that controls noncanonical NF-κB signaling, in an ATP/ADP-based kinase assay. Consistent with this, plumbagin inhibited processing of NF-κB2 p100 to p52 in the progenitor cells of both OCs and osteoblasts (OBs). Interestingly, plumbagin not only inhibited OC but also stimulated OB differentiation *in vitro*. Importantly, plumbagin prevented trabecular bone loss in ovariectomized mice. This was associated with decreased OC surfaces on trabecular surface and increased parameters of OBs, including OB surface on trabecular surface, bone formation rate, and level of serum osteocalcin, compared to vehicle-treated mice. In summary, we conclude that plumbagin is a NF-κB-inducing kinase inhibitor with dual anabolic and antiresorptive effects on bone and could represent a new class of agent for the prevention and treatment of osteoporosis.

Osteoporosis is a major aging disease characterized by decreased bone mass and strength, resulting in increased fracture risk. About 44 million people in the U.S. have a low bone mineral density, and 10 millions of them are osteoporotic (1). Elderly patients with osteoporotic fractures develop complications, including pneumonia and deep vein thrombosis due to prolonged bed rest, and up to 25% of them die within the first 6 months after fracture. Osteoporosis is caused by an imbalanced bone remodeling, a process involving in enhanced bone resorption and relatively reduced bone formation mediated by osteoclast (OC) and osteoblast (OB), respectively (1). Two classes of drugs are available to treat osteoporosis: antiresorptive and anabolic. Antiresorptive agents, including bisphosphonates (BPs) and denosumab (receptor activator of nuclear factor kappa-B ligand [RANKL] monoclonal antibody), inhibit bone resorption to increase bone mass. However, they also inhibit bone formation (2, 3) and only reduce the rate of osteoporotic fracture by ∼50% (4). Reports of atypical femoral fractures (5, 6) and jawbone necrosis (7) caused by these antiresorptive agents have reduced patients’ willingness to take them. In addition, one special concern is that denosumab discontinuation is associated with a subsequent profound increase of bone turnover above pretreatment values because of a vast increase of OC number and activity, resulting in a risk of multiple vertebral fractures (8, 9). The anabolic agents include teriparatide (N terminus 34 amino acid of parathyroid hormone) and newly U.S. Food and Drug Administration-approved abaloparatide (a parathyroid hormone-related protein analog) for the treatment of osteoporosis by stimulating new bone formation (10). However, they are limited to use for 1 to 2 years due to long-term biosafety concerns for the potential to induce osteosarcoma (11, 12, 13, 14). In addition, effect of the anabolic agents to increase bone mass is transient, and discontinuing these agents abruptly decrease bone mineral density (15) due to increased bone resorption (16). Thus, the use of these anabolic treatment must be followed by antiresorptive agents (10). Romosozumab, a sclerostin monoclonal Ab, has dual effect to increase bone formation early (and transiently) while inhibiting bone resorption persistently to treat osteoporosis (17). However, it can increase the risk of severe side effects including myocardial infarction, stroke, and cardiovascular death (13). Thus, romosozumab is only recommended for treatment of severe osteoporosis for up to 1 year (18). Development of new class of antiresorptive and anabolic agents would meet a great unmet need for the prevention and treatment of osteoporosis.

We have explored if a small molecular NF-κB-inducing kinase (NIK) inhibitor is able to prevent osteoporosis because (1) NF-κB family of transcriptional factors plays a central role in OC differentiation by sequentially activating c-Fos followed by NFATc1 (19, 20), the two other transcriptional factors critical for OC differentiation; (2) NIK, the key kinase that activates noncanonical NF-κB signaling by processing NF-κB2 p100 into p52, controls RANKL-induced osteoclastogenesis and the development of peripheral lymph nodes and B and T lymphocytes, and thus NIK deficiency results in the complete resistance of the mice to antigen-induced arthritis (21); and (3) NIK also controls canonical NF-κB activation (22). A number of small molecular NIK inhibitors have been identified (23, 24, 25), some of which are able to inhibit immune response (23, 24). It has reported that a NIK inhibitor, Cpd33, prevents bone loss in ovariectomized mice by inhibiting bone resorption (26). However, antiresorptive agents have been widely used to treat osteoporosis. It is more interesting to identify new NIK inhibitors with dual antiresorptive and anabolic effects for the treatment of osteoporosis.

A small molecular compound, 5-hydroxy-2-methyl-1,4-naphthoquinone, was identified to inhibit NIK activity by Mortier (25). It is identical to plumbagin, which has been previously reported to inhibit OC formation and to attenuate breast cancer–induced osteolysis (27, 28). We report here that plumbagin not only inhibits OC formation but also stimulates OB differentiation by inhibiting NIK activity and the subsequent NF-κB2 p100 processing and thus efficiently prevents osteoporosis in ovariectomized mice.

## Results

### Plumbagin inhibits NIK activity and NF-κB p100 processing

**Figure 1:**
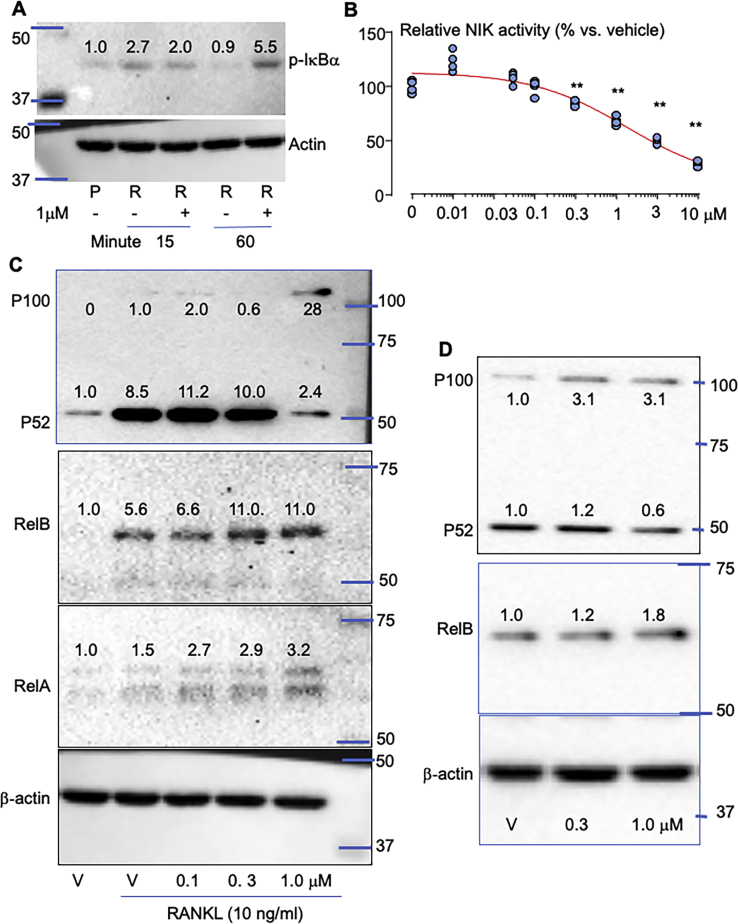
**Plumbagin inhibits NIK activity and NF-κB2 p100 processing.***A*, bone marrow cells from C57BL6 mice were cultured with M-CSF for 3 days to expand macrophages. The cells were starved for 4 h followed by treatment with RANKL (R) plus vehicle or plumbagin (1 μM) for 15 and 60 min, respectively. Phosphorylated (p)-IκBα was tested by Western blot (WB). *B*, NIK activity was tested in the presence of indicated dose of plumbagin using ADP-Glo Kinase Assay kit, four repeats each dose (one sample each dot). ∗∗*p* < 0.01 *versus* vehicle (0). *C*, macrophages, generated as in (*A*), were treated with RANKL in the presence of vehicle or plumbagin for 8 h. Cell lysates were used to test protein levels of p100/p52, RelB, RelA, and β-actin by WB. *D*, CH310T1/2 mouse MSCs were treated with vehicle (V) or plumbagin for 8 h. P100/p52, RelB, and β-actin proteins in the cell lysate were tested by WB. Each WB was repeated three times with similar results. NIK, NF-κB-inducing kinase; RANKL, receptor activator of nuclear factor kappa-B ligand.

NF-κB family of transcription factors are activated through canonical and noncanonical signaling pathways. The key initial event in canonical NF-κB signaling is the phosphorylation of IκBα by a trimeric IκB kinase complex to release RelA:p50 dimers, allowing them translocate to the nucleus to activate target genes (20, 29). It has been published that plumbagin inhibits RANKL-induced OC formation and canonical NF-κB activation by preventing IκBα phosphorylation and degradation (27). However, the role of the key transcriptional factor p65 in canonical NF-κB pathway is to promote OC progenitor cell (OCP) proliferation, but it does not affect terminal OC formation (30). Consistent with the published result (31), RANKL markedly induced IκBα phosphorylation within 15 min (Fig. 1*A*). However, addition of plumbagin did not change RANKL-induced IκBα phosphorylation at this time point (Fig. 1*A*). After 1 h, protein level of phosphorylated IκBα in RANKL-treated cells went back to the basal level while addition of plumbagin maintained RANKL-induced IκBα phosphorylation at high level after 1 h (Fig. 1*A*). These findings suggest that plumbagin does not mainly regulate RANKL-induced canonical NF-κB activation but would prolong RANKL-induced canonical NF-κB activation.

It is not known if plumbagin inhibits RANKL-induced OC formation *via* regulating noncanonical NF-κB signaling. The key event for noncanonical NF-κB signaling is the processing of p100 to p52 by NIK (32). We were unable to test NIK protein by Western blot because the basal level of NIK protein is very low under normal physiological conditions (33). We used ADP-Glo Kinase Assay kit to test the effect of plumbagin on NIK activity and found that plumbagin effectively inhibited NIK activity in a dose dependent-manner and the IC50 to inhibit NIK activity is 3.0 μM (Fig. 1*B*). We then tested if plumbagin prevents RANKL-induced p100 processing because our previous findings indicate that RANKL efficiently processes p100 to p52 to promote OC differentiation (34). As expected (34), RANKL markedly increased p52 protein level (8.5-folds) while p100 protein level was kept low (Fig. 1*C*), indicating that RANKL efficiently processes p100 to p52 in OCPs. Protein level of p100 in RANKL-treated cells was slightly increased compared to that in vehicle-treated OCPs because RANKL stimulates the mRNA expression of p100 precursor (34). Plumbagin (1.0 μM) sharply increased p100 protein level (28-folds) while reduced p52 protein level (from 10- to 2.4-folds) in RANKL-treated OCPs although lower dose (0.1 and 0.3 μM) of plumbagin did not markedly change the protein levels of both p100 and p52 (Fig. 1*C*). These findings suggest that plumbagin inhibits RANKL-induced activation of noncanonical NF-κB (processing of p100 to p52) in OCPs. RelB forms heterodimer with p100/p52, and therefore RANKL markedly increased RelB protein level while higher dose (0.3 and 1 μM) of plumbagin slightly increased RANKL-induced RelB protein levels in OCPs (2-folds, Fig. 1*C*). RANKL also slightly increased RelA protein level (1.5-folds), and plumbagin from 0.1 to 1 μM further increased RANKL-induced RelA protein levels by 3-folds in OCPs (Fig. 1*C*). Similarly, 0.3 and 1 μM of plumbagin increased p100 protein levels by 3-folds and 1 μM of it decreased p52 protein levels by 0.6-fold in mouse C3H10T1/2 mesenchymal stem cells (MSCs) (Fig. 1*D*), suggesting that it also inhibits noncanonical NF-κB activation in MSCs. Of note, plumbagin may also slightly increased RelB protein levels in C3H10T1/2 MSCs (Fig. 1*D*).

### Plumbagin not only inhibits OC formation but also stimulates OB differentiation *in vitro*

**Figure 2:**
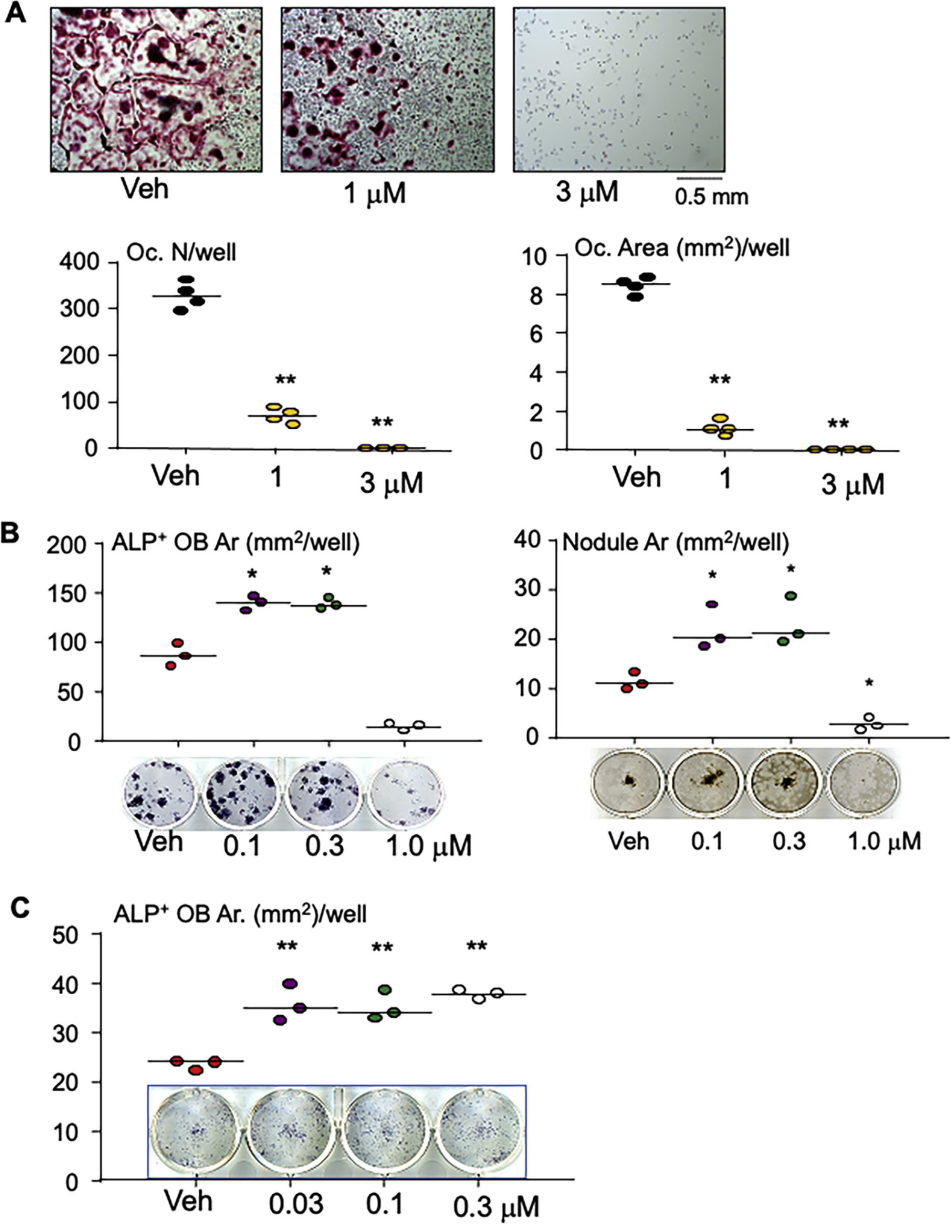
**Plumbagin inhibits OC and promotes OB differentiation.***A*, bone marrow cells from C57Bl6 mice were cultured with M-CSF for 2 days to generate macrophages, which were then treated with RANKL plus indicated doses of plumbagin in the presence of M-CSF for 3 days. Cells were stained with TRAP activity (*upper panel*) to evaluate OC number and area (Ar). N= 4 per group. ∗∗*p* < 0.01 *versus* vehicle (Veh). *B*, bone marrow cells from 3-month-old C57Bl6 mice were cultured with Minimum Essential Medium alpha containing 15% FBS to expand stromal cells, which were then induced for OB differentiation in the presence of indicated dose of plumbagin for 6 and 14 days, respectively. ALP staining (6 days) and Von Kossa staining (14 days) were performed to measure the area (Ar.) of ALP^+^ cells and mineralized nodule, respectively. N = 3 per group. ∗∗*p* < 0.01 *versus* Veh. *C*, BdMPCs were induced for OB differentiation in the presence of indicated dose of plumbagin for 6 days. ALP staining was performed to measure ALP^+^ cell area (Ar.). n = 3/group. ∗*p* < 0.05 and ∗∗*p* < 0.01 *versus* Veh. ALP, alkaline phosphatase; BdMPCs, bone-derived mesenchymal progenitor cells; OB, osteoblast; OC, osteoclast; RANKL, receptor activator of nuclear factor kappa-B ligand; TRAP, tartrate-resistant acid phosphatase.

Consistent with the published results, plumbagin inhibited RANKL-induced OC formation, starting around 1 μM (Fig. 2*A*). Since antiresorptive agents, including a variety of BPs and denosumab, are widely used in clinic to treat osteoporosis, it is more interesting to find out agents with anabolic effect. Thus, we tested if plumbagin can stimulate OB differentiation. The BM cells freshly flushed out from femoral and tibial shaft were cultured to expand BM stromal cells followed by induction of OB differentiation in the presence of plumbagin. We found that 0.1 and 0.3 μM of plumbagin significantly increased alkaline phosphatase (ALP)^+^ OB differentiation but higher dose (1 μM) inhibited it from BM stromal cells (Fig. 2*B* left panel). Similarly, lower dose (0.1 and 0.3 μM) stimulated mineralized nodule formation from BM stromal cells (Fig. 2*B* right panel). Primary mouse BM stromal cells contain large number of macrophages, which help OB differentiation and mineralization (35). Therefore, we also used pure bone-derived mesenchymal progenitor cells (BdMPCs) (36) to further test the effect of this compound on OB differentiation. The results indicated that plumbagin ranging from 0.03 to 0.3 μM significantly increased ALP^+^ OB area (Fig. 2*C*), confirming that it stimulates OB differentiation directly.

### Overexpression of NF-κB p100 enhances plumbagin stimulation of OB differentiation

**Figure 3:**
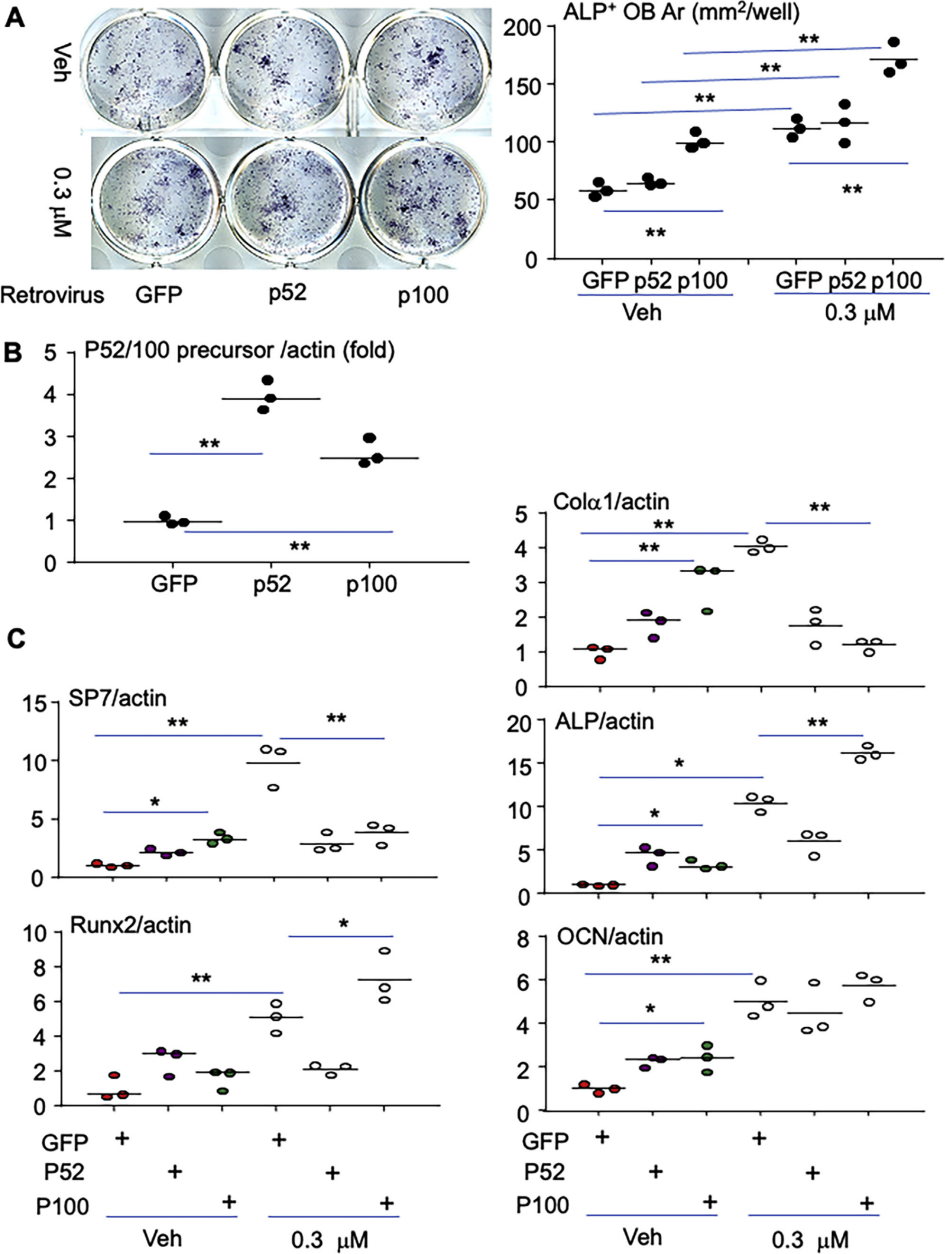
**Overexpression of NF-κB2 p100 enhances the effect of plumbagin to increase OB differentiation.** BdMPCs from WT mice in 12-well plates were incubated with 1/4 volume of pMX-GFP, pMX-p52, or pMX-p100 retroviral supernatants in the presence of 2 μg/ml of polybrene. *A*, after 24 h, the infected cells were begun to treat with vehicle (Veh) or 0.3 μM of plumbagin for 6 days. The cells were fixed with 10% neutral buffered formalin followed by ALP staining to measure the area (Ar.) of ALP^+^ cells, as in Fig. 2*C*, n = 3. *B*, the expression of p100/p52 mRNA expression in GFP, p52, or p100 infected cells was tested by real-time PCR, N = 3, ∗*p* < 0.05, ∗∗*p* < 0.01. *C*, the infected cells in 60-mm dishes were treated with 0.3 μM of plumbagin for 6 days. Total RNAs were extracted to test mRNA expression of OB marker genes, including Colα1, ALP, osteocalcin (OCN), SP7, and Runx2, normalized by β-actin. N = 3. ∗*p* < 0.05, ∗∗*p* < 0.01. ALP, alkaline phosphatase; BdMPCs, bone-derived mesenchymal progenitor cells; OB, osteoblast.

It is known that NIK controls RANKL-induced osteoclastogenesis (21) and constitutive activation of NIK in OB lineage cells causes a high bone turnover phenotype (both bone resorption and formation are increased) (37). To further examine if plumbagin stimulates OB differentiation through inhibiting NIK processing NF-κB2 p100, we investigated the effect of overexpression of p52 or p100 on plumbagin-induced OB differentiation. As expected, in GFP control retrovirus infected cells, 0.3 μM plumbagin significantly increased ALP^+^ OB area (Fig. 3*A*). Importantly, overexpression of p100 but not p52 further enhanced the effect of plumbagin to increase OB differentiation (Fig. 3*A*). These findings are consistent with our hypothesis that inhibition of NIK processing NF-κB2 p100 (p100 accumulation) enhances OB differentiation. The successful overexpression of p52 or p100 was confirmed by the real-time PCR showing that either p52 or p100 retroviral infection increased p100/p52 precursor mRNA expression (Fig. 3*B*) (19).

Colα1, ALP, and OCIN are the early-, middle-, and late-stage markers of OB differentiation, representing proliferation, differentiation, and mineralization of OBs (38, 39, 40), respectively. Osterix/Sp7 is a critical transcription factor downstream from Runx2 for OB differentiation and maturation by regulating the expansion of an early osteoblastic pool derived from MPCs (41, 42). However, Sp7 inhibits late-stage OB differentiation (43). We further evaluated the effect of plumbagin on OB differentiation by testing the expression of these OB marker genes during OB differentiation from MPCs infected with GFP, p52, or p100 retrovirus. Similar to the functional OB differentiation assay (Fig. 3*A*), overexpression of p100 but not p52 significantly increased mRNA expression of Sp7, Colα1, ALP, and OCIN in the cells without plumbagin treatment (Fig. 3*C*). Plumbagin alone (in GFP-infected cells) increased mRNA expression of Sp7, Runx2, Colα1, ALP and Sp7, and OCIN (Fig. 3*C*). Interestingly, when p100 was overexpressed, plumbagin decreased the expression of early OB markers, Colα1 and Sp7, and increased the middle-stage markers, Runx2 and ALP, but did not change OCIN mRNA expression, in MPCs (Fig. 3*C*). These findings suggest that plumbagin promotes middle-stage and late-stage differentiation and maturation of OBs from MPCs with high level of p100 expression (NIK inhibition).

### Plumbagin prevents trabecular bone loss in ovariectomized mice

**Figure 4:**
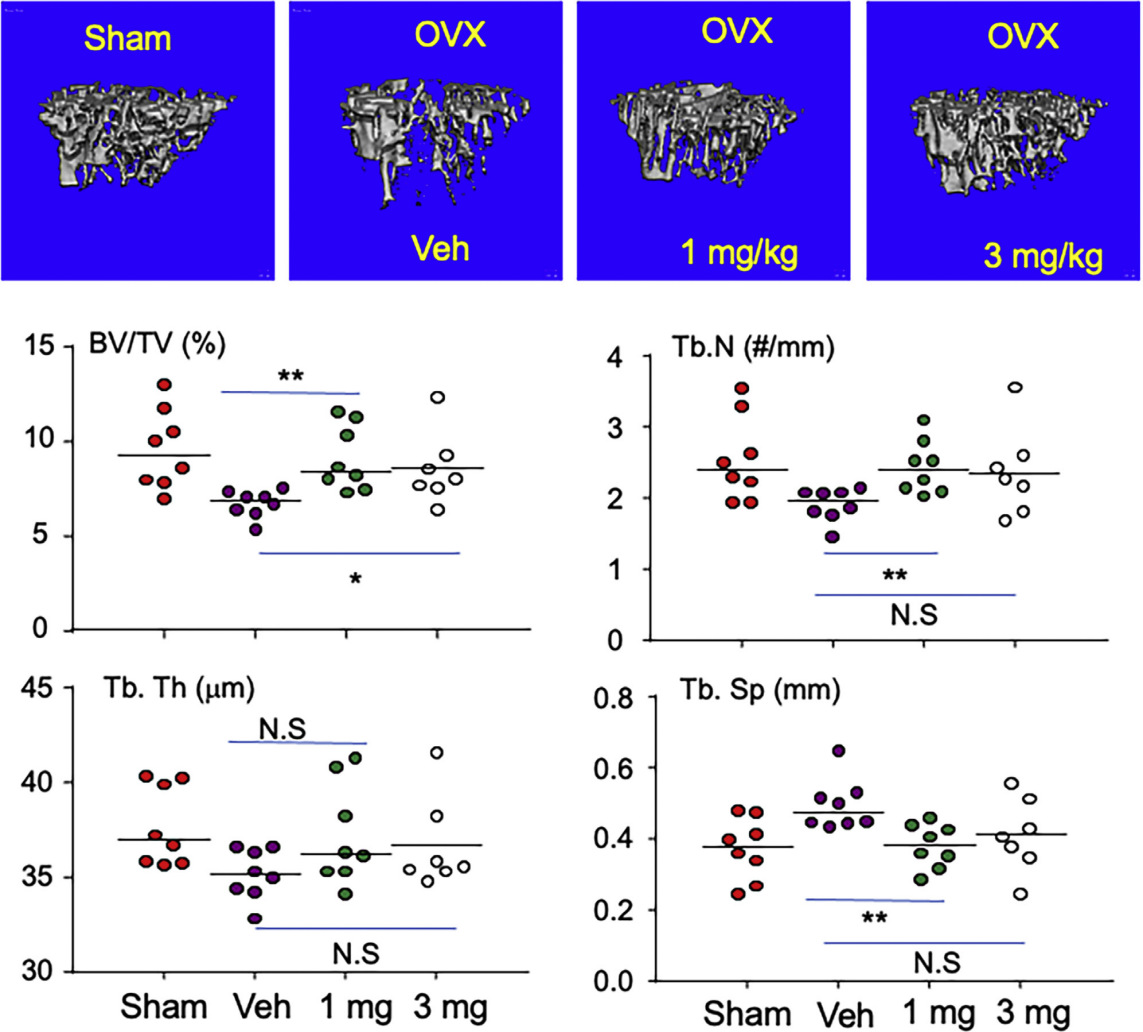
**Plumbagin prevents OVX-induced trabecular bone loss in mice.** Three-month-old female C57Bl6 mice were performed sham or OVX. From the third day, the OVX mice were treated with vehicle (V), 1 or 3 mg/kg of plumbagin, 5 times/week, for 5 weeks, eight mice per group. After the mice were sacrificed, the right tibia were scanned with micro-CT (one leg was lost due to its breaking during tissue harvesting in 3 mg treated group) to analyze the structural bone parameters: bone mass (BV/TV), trabecular number (Tb.N), trabecular thickness (Tb. Th), and trabecular separation (Tb.Sp). ∗∗*p* < 0.01, ∗*p* < 0.05, NS = no statistical significance; OVX, ovariectomy.

We performed ovariectomy (OVX) or sham surgery in 3-month-old female C57Bl6 mice. From the third day, the OVX mice were treated with vehicle, 1, and 3 mg/kg of plumbagin for 5 weeks. As expected, compared to the mice with sham surgery, OVX significantly reduced trabecular bone volume/tissue volume (BV/TV), characterized by decreased trabecular number (Tb.N) and trabecular thickness (Tb.Th) in the tibia (Fig. 4), evaluated by μCT. Importantly, 1 mg/kg and 3 mg/kg of plumbagin significantly increased trabecular BV/TV compared to vehicle in OVX mice, and their tibial BV/TV in OVX mice treated with both dose of plumbagin were maintained to the level of sham mice (Fig. 4). The increased bone mass in plumbagin-treated OVX mice was likely caused by increased Tb. N (Fig. 4). However, 3 mg/kg of plumbagin did not further increase BV/TV compared to 1 mg/kg (Fig. 4).

### Plumbagin inhibits bone resorption and increases bone formation in OVX mice

**Figure 5:**
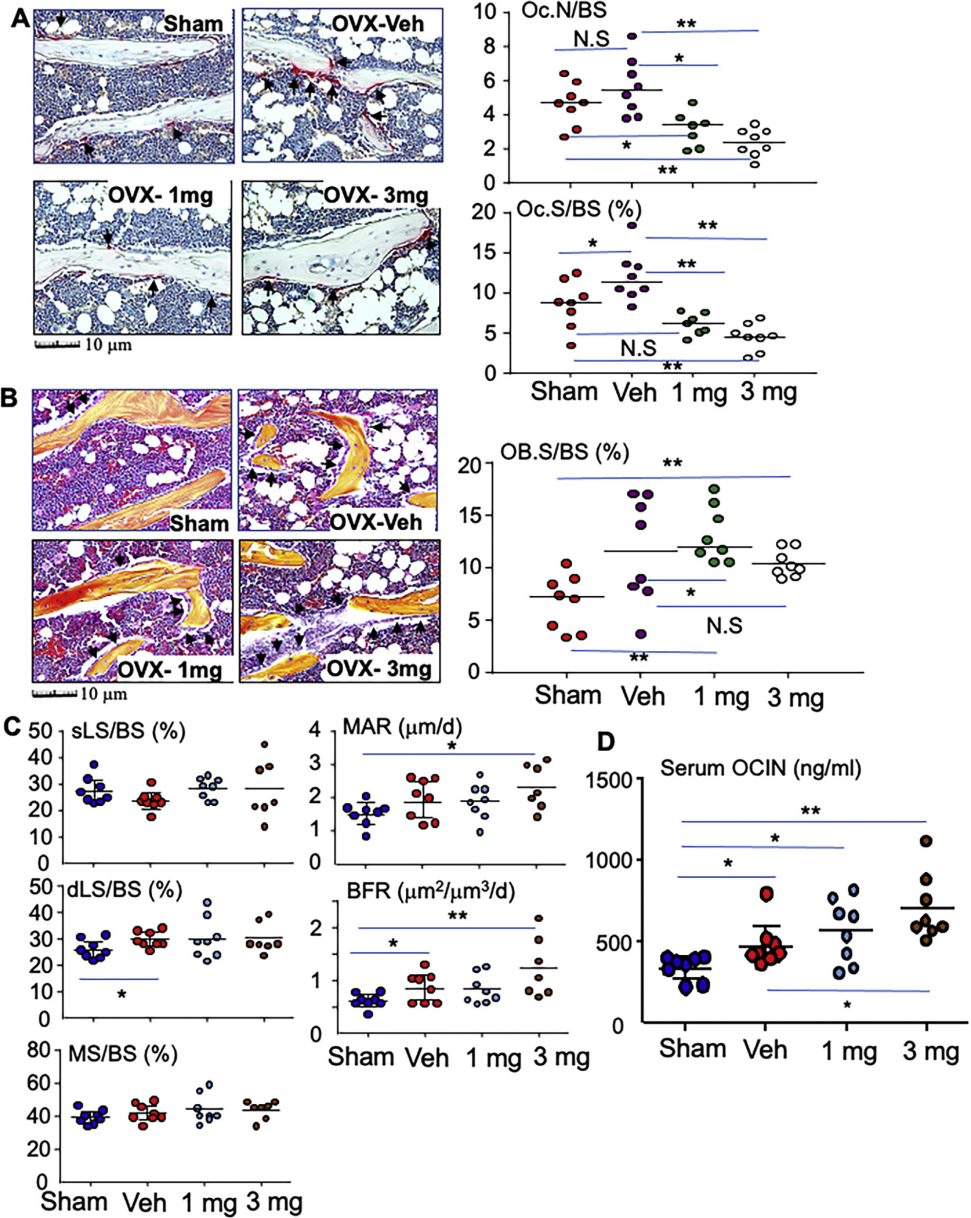
**Plumbagin inhibits bone resorption while it maintains the increased bone formation in OVX mice.** A, representative images of TRAP-stained left tibial slices (*upper panel*) from mice, as in Figure.4, and quantification of the surface and number of OCs that actively resorb bone on the trabecular surface (*lower panel*). *Black arrows* show TRAP-stained OCs that actively resorb bone on the trabecular surface with resorptive lacunae. ∗*p* < 0.05, ∗∗*p* < 0.01, NS = no statistical significance. *B*, representative images of H&E stained left tibial slices (*upper panel*) and quantification of OB surface (*lower panel*) from mice as above (*A*). *Black arrows* show OBs on trabecular bone surface (BS). ∗*p* < 0.05, ∗∗*p* < 0.01, NS=no statistical significance. *C*, the right tibiae, after micro-CT scanning as in Figure.4, were processed as plastic sections to quantify the dynamic bone formation parameters: single labeling surface (sLS/BS), double labeling surface (dLS/BS), mineralizing surface (MS/BS), mineral apposition rate (MAR), and bone formation rate (BFR). ∗∗*p* < 0.01, ∗*p* < 0.05, NS = no statistical significance. *D*, serum from all mice were used to test bone formation marker, osteocalcin (OCN), by ELISA. ∗*p* < 0.05, ∗∗*p* < 0.01. OB, osteoblast; OC, osteoclast; OVX, ovariectomy; TRAP, tartrate-resistant acid phosphatase.

Postmenopausal osteoporosis is characteristic by high bone turnover in human (44, 45) and rodent (46), namely both bone resorption and formation are increased while bone resorption is more than formation to result in accelerated bone loss. Consistent with this, OC surface on trabecular surface was significantly increased in the tibia of OVX mice compared to that of sham mice (Fig. 5*A*). Interestingly, both doses of plumbagin significantly reduced OC surface on trabecular surface in OVX mice compared to vehicle (Fig. 5*A*). Of note, the tibial OB surface had trend to increase in OVX mice but did not show statistic difference compared to that in sham mice (Fig. 5*B*). In contrast, OB surface in the tibia from mice treated with either dose of plumbagin was significantly higher than that from sham mice although it did not show statistic difference compared to that of OVX mice treated with vehicle (Fig. 5*B*) because OVX had trend to elevate OB surface in the OVX mice compared to sham mice.

The dynamic parameters of bone formation were evaluated on plastic sections of the tibia. The double labeling surface in OVX mice was significantly increased compared to that in sham mice (Fig. 5*C*). This is consistent with the finding that the OVX mice have trend to increase OB surface (Fig. 5*B*). However, the bone formation rate (BFR) did not have statistical difference between the OVX and sham mice (Fig. 5*C*) because OVX mice had trend to decrease single labeling surface although they had trend to increase mineral apposition rate (MAR) compared to sham mice (Fig. 5*C*). This is similar to the published results that the osteoporotic model in OVX mice does not have increased BFR although they generally have accelerated bone turnover, increased both markers of bone resorption and bone formation such as OB surface (47). Interestingly, the OVX mice treated with 3 mg/kg of plumbagin had significantly increased BFR due to increased double labeling surface and MAR compared to the sham mice, but these parameters in OVX mice treated with 3 mg/kg of plumbagin did not have significant difference compared to that in vehicle-treated OVX mice because OVX itself had increased BFR compared to sham mice (Fig. 5*C*). However, increased BFR in OVX mice treated with 1 mg/kg of plumbagin did not show statistical difference compared to sham mice. Of note, level of serum OCIN in OVX mice treated with vehicle was significantly higher than that in sham mice (Fig. 5*D*). Importantly, level of serum OCIN in OVX mice treated with 3 mg/kg of plumbagin was significantly higher than that not only in sham mice but also in OVX mice treated with vehicle (Fig. 5*D*). However, 1 mg/kg of plumbagin did not increase serum OCIN in OVX mice, but its level in OVX mice treated with 1 mg of plumbagin was higher than that in sham mice (Fig. 5*D*). These findings suggest that plumbagin maintains the increased bone formation in the OVX mice.

## Discussion

In this report, we have established a novel concept that a small molecular NIK inhibitor exhibits dual anabolic and antiresorptive effects to bone and thus could be used for the prevention and treatment of osteoporosis. Although a NIK inhibitor, Cpd33, has been reported to be an antiresorptive agent (26) and plumbagin inhibits OC formation (27, 28), we first identified that plumbagin is a NIK inhibitor with dual anabolic and antiresorptive effects to bone. It dose-dependently inhibits NIK activity in the *in vitro* ATP/ADP kinase assay system (Fig. 1*B*) and inhibits the processing of noncanonical NF-κB2 p100 to p52 in the progenitor cells of both OCs and OBs (Fig. 1, *C* and *D*). Particularly, it not only inhibits RANKL-induced OC formation (Fig. 2*A*), as reported previously (27), but also stimulates OB differentiation from bone marrow (BM) stromal cells and pure MSCs *in vitro* (Fig. 2, *B* and *C*). Importantly, plumbagin efficiently prevents OVX-induced bone loss (Fig. 4), associated with reduced OC number and surface while increased levels of OB surface and serum OCIN, and maintains high levels of MAR and BFR (Fig. 5).

It has long been known that NIK processes noncanonical NF-κB2 p100 to p52 to control OC differentiation in inflammatory arthritis (21). In the unstimulated cells, NIK is kept at low level (33) because it undergoes constitutive ubiquitination and proteasomal degradation by interacting with TRAF3 and cIAP proteins (48, 49). RANKL induces TRAF3 lysosomal degradation in a TRAF6- independent manner in OC precursors (50, 51); as a result, NIK is accumulated in the cells to process p100 to p52 and to result in subsequent OC differentiation (34, 50). Consistent with this, both OC differentiation and function are enhanced when NIK lacks TRAF3 binding domain (52). Plumbagin has been known to attenuate metastatic cancer–induced osteolysis by inhibiting OC formation (27, 28). However, to our knowledge, this is the first report showing that plumbagin inhibits NIK activity and subsequent p100 processing to attenuate RANKL-induced OC formation.

This report also demonstrates a first evidence that a small molecular NIK inhibitor can stimulate OB differentiation. Plumbagin inhibits NIK activity (Fig. 1*B*) to result in the accumulation of p100 protein in MPCs (Fig. 1, *C* and *D*) and thus increases ALP+ OB differentiation from mouse BM stromal cells and BdMPCs (Fig. 2, *B* and *C*). Importantly, plumbagin significantly increases the parameters of bone formation, including circulating bone formation marker, OCIN, and dynamic BFR, in OVX mice (Fig. 5). These findings are consistent with the reports that accumulation of unprocessed NF-κB2 p100 enhances osteoblastic differentiation (53) and deletion of p100, but retaining a functional p52, results in osteopenia owing to increased OC activity and impaired OB parameters in mice (54). RelB, the partner of p100/p52, is also a downstream protein of NIK (55). Genetic deletion of RelB results in increased bone mass associated with increased OB (36) and decreased stimulatory OC differentiation (55). Consistent with this, deletion of RelB prevents the osteopenia phenotype in p100^−/−^ mice associated with increased OB surfaces (54). NIK also involves in activation of canonical NF-κB pathway (22), which is consistent with our findings that plumbagin slightly increases protein levels of RelA (Fig. 2, *C* and *D*). However, plumbagin-stimulated OB differentiation is unlikely *via* regulating canonical NF-κB because canonical NF-κB signaling inhibits early-stage and late-stage OB differentiation (56, 57, 58, 59), although it also stimulates MSC proliferation (60).

Bone remodeling is a coupling event of bone resorption and formation, not only in normal but also in pathological conditions. In a normal adult, the amount of lacunae resorbed by OCs is equally repaired by OBs. In postmenopausal osteoporosis, bone turnover is accelerated, and both bone resorption and formation are increased (45, 61, 62) but bone formation is less than resorption to result in bone loss (63). Another example for the coupling of bone formation to resorption is that in addition to increase bone resorption directly independent of RANKL-TRAF6 axis (34, 51, 64) and indirectly by stimulating RANKL production (65), TNF-α exhibits anabolic effect to bone through its polarized macrophage to slow down bone loss in rheumatoid arthritis (35). There is a possibility that plumbagin inhibition of bone resorption causes secondarily increased bone formation, like transplantation of hematopoietic stem cells from *oc/oc* osteopetrotic mice with defective acid secretion into irradiated normal mice causes decreased bone resorption coupled with temporarily increased bone formation (66). Another aspect of bone formation coupling resorption is that BPs (2, 16) and denosumab (3) also inhibit bone formation. Thus, plumbagin, which inhibits bone resorption while simultaneously maintains high level of bone formation in OVX mice (Fig. 5), appears to offer advantage over the use of BPs that inhibit both bone resorption and formation (2, 3). It is important to further test if plumbagin might be able to avoid the side effects of BPs such as atypical fracture (67, 68) and jaw bone necrosis (7, 69). Plumbagin is also different from anabolic agent, teriparatide, that stimulates bone resorption followed by longer bone formation to increase bone mass (70).

Of note is that higher dose (1.0 μM) of plumbagin inhibits OC differentiation effectively (Fig. 2*A*), but this dose also reduces ALP^+^ OB differentiation from BM stromal cells to the basal level (vehicle treated cells) (Fig. 2*B*), suggesting that high dose of plumbagin could inhibit bone formation although the potential to inhibit bone resorption is enhanced. We also noticed that plumbagin slightly increases protein level of RelA in OCPs (Fig. 1*C*), which are known to stimulate OC differentiation (71) by promoting OCP proliferation (30). It is not known if plumbagin activation of canonical NF-κB signaling could secondarily attenuate its effect to inhibit bone resorption. In particular, higher dose (3 mg/kg) of plumbagin does not appear to offer advantage over the use of lower dose (1 mg/kg) to increase bone mass (Fig. 4). Therefore, the optimal (lowest effective) dose of plumbagin to increase bone mass by stimulating bone formation while effectively inhibiting bone resorption should be further investigated in future.

In past three decades, many small molecule compounds, targeting IκBα, p65, and IκB kinase complex, have been developed to inhibit canonical NF-κB activation (29, 72). Some of them have been studied in clinical trials, aiming to treat malignant tumors, inflammation, and autoimmune diseases (72). But for none of them clinical trials have been performed to treat osteoporosis. In addition, numerous small molecule compounds, targeting cytokine receptors, receptor adaptors, kinases, E3 ubiquitin ligase, proteasome, and deubiquitination, are able to inhibit NF-κB activation (72). In recent years, several NIK inhibitors have been developed (23, 24, 73, 74). One of them, Cpd33, attenuates bone loss in OVX mice by inhibiting bone resorption but not stimulating bone formation (26). Currently, we cannot interpret the discrepancy between plumbagin and Cpd33 for the effect on OB differentiation. It is worth to further test if other NIK inhibitors also exhibit dual antiresorptive and anabolic effects in order to choose those with better effects while low toxic profiles for the treatment of bone diseases with bone loss.

In summary, plumbagin inhibits OC and stimulates OB differentiation through inhibiting NIK activity *in vitro*, and importantly, it prevents trabecular bone loss associated with decreased OCs, while increased OBs and BFR in OVX mice. However, OVX-induced osteoporosis is characterized by high bone turnover, in which bone formation parameters have been increased. It will be necessary to use other osteoporosis model with low bone turnover, for example age-related osteoporosis, to further test the effect of plumbagin to increase bone formation.

## Experimental procedures

### Reagents

Recombinant murine macrophage colony-stimulating factor (M-CSF) and RANKL were purchased from R&D Systems. Antibodies against p100/p52 (Cat# 4882s) and p-IκBα (Cat# 9246s) were purchased from Cell Signaling, p65 (Cat# sc-372), and RelB (Cat# sc-226) antibodies were from Santa Cruz, and β-actin antibody was purchased from Sigma (Cat# A5441). The compound 5-hydroxy-2-methyl-1,4-naphthoquinone (plumbagin) was purchased from MolPort Inc.

### Animal surgery and drug administration

Forty 3-month-old female C57Bl6 mice, purchased from Jax lab, were used to evaluate the effect of plumbagin on the prevention of osteoporosis induced by OVX. Eight mice were randomly divided into sham and 24 mice in OVX group, which were performed sham and OVX surgery, respectively. In the second day, the ovariectomized mice were randomly divided into three groups: vehicle, 1 and 3 mg/kg of plumbagin. From the third day, the OVX mice were treated with vehicle or plumbagin, in the volume of 0.1 ml per 10 g body weight, through I.P. injection, once a day for 5 weeks. Mice were given injections of calcein (10 mg/Kg) 5 and 1 day before sacrifice following our standard protocol (34, 36). All animal experimental protocols were approved by the University of Rochester Committee for Animal Resources, and all methods were carried out in accordance with the guidelines and regulations of the American Veterinary Medical Association.

### Micro-CT evaluation

The right legs were fixed in 10% neutral buffered formalin for 48 h and were transferred to 70% ethanol at 4 °C for storage. The tibia was scanned using a vivaCT 40 instrument (Scanco Medical) at a voxel size of 7 μm, 50 kVp, 144 μA, and 800 ms integration time. The machine was set at a threshold of 220 to distinguish bone from soft tissues. Cancellous bone was assessed in 300 transverse slices to determine bone volume (BV/TV, %), Tb.Th (μm), Tb.N (#/mm), and trabecular separation (μm) according to standard guidelines (75).

### Bone histomorphometric analysis

Left legs were fixed in 10% neutral buffered formalin for 48 h, decalcified 3 weeks using 10% EDTA at 4 °C, and processed, embedded in paraffin. We selected a slice at the center of the tibial bone from three levels of continuously cut slices, 30-μm interval between the two levels, for bone histomorphometric analysis. Briefly, the paraffin blocks were trimmed to the level adjacent to the center of the tibia, observed under microscope, and begun to collect 3- μm thick slices as first level. The block was continuously cut to discard 30 μm-thick tissue and then 3-μm thick slices were collected again as second level. The above procedure was repeated to discard 30-μm-thick tissue and 3-μm-thick slices were collected as third level. The first slice of each level was performed H&E staining. The H&E–stained slices from each block was compared under microscope to select the slice with fewest trabeculae which represents the center of the bone and is comparable among different mice. The second slice from the selected level with fewest trabeculae was performed tartrate-resistant acid phosphatase (TRAP) staining. The selected H&E- and TRAP-stained slices were respectively given a number in order to evaluate histomorphometric parameters blindly. The structural trabecular bone parameters, BV/TV (%), Tb.Th (μm), Tb.N (#/mm), trabecular separation (μm), and OB surface were measured on the tibia of H&E stained slices, and OC parameters were measured on TRAP-stained tibia using an OsteoMeasure Image Analysis System (Osteometrics) (34, 36) following the instruction of ASBMR Histomorphometry Nomenclature Committee (76).

The right legs, after micro-CT scanning, were dehydrated by 95% and 100% ethanol serially. The samples were then dipped in 1 ml L.R. White Embedding Medium (Polysciences Inc) in a glass bottle in the air for 2 h. The LR White Medium was changed with fresh one, and the bottles were transferred into a vacuum tank for 48 h, during which the L.R. White Medium was changed once. A bone sample was placed in a mold filled with fresh L.R. White Medium, which was then put in a 55 °C oven to solidify the medium. A block was wrapped with aluminum foil in the air at room temperature for continuous solidification for at least 1 week. Three-micrometer-thick plastic sections were cut using a carbide steal knife on a Shandon Microtome. The sections at the center of the bone were collected, following the general instruction for paraffin block cutting, for the evaluation of dynamic parameters of bone formation using an OsteoMeasure Image Analysis System (Osteometrics) (34, 36) following the instruction of ASBMR Histomorphometry Nomenclature Committee (76).

### *In vitro* assay for osteoclastogenesis

The culture procedure was modified from our previous reports (51, 77, 78). Briefly, cut open both end of each femur or tibia to expose the marrow cavity, flush out BM with 10 ml of Minimum Essential Medium alpha containing 2% FBS by using 21-gauge needle, and pass the cells through a 21-G needle three times to make single cell suspensions. The cells were incubated in NH_4_Cl solution for 15 min at room temperature to lyse red blood cells. 5 × 10^4^ cells was seeded in a well of 96-well plates with 5 ng/ml M-CSF for 2 days. Then RANKL (10 ng/ml) and different inhibitors were added to the culture for additional 2 to 3 days when mature OCs typically are observed under inverted microscopy. The cells were then fixed with 10% neutral, phosphate-buffered formalin for 10 min and stained for TRAP activity. TRAP^+^ cells with three or more nuclei were considered to be mature OCs.

### *In vitro* OB differentiation assay

BM (1 × 10^6^) cells from WT mice were seeded in 12-well plates with Minimum Essential Medium alpha containing 15% FBS for 5 days followed by induction of OB differentiation with 25 μg/ml ascorbic acid and 5 mM β-glycerophosphate (36). The cells were fixed with 10% neutral buffered formalin followed by ALP staining after 7 days and Von Kossa staining after 14 days to measure the area (Ar.) of ALP^+^ cells and mineralized nodule, respectively. Similarly, the BdMPCs from the WT mice (36) was used to test the effect of the compounds on OB differentiation. Briefly, BdMPCs were seeded in 12-well plates, 1 × 10^4^/well. From the second day, the cells were induced for OB differentiation, as above, in the presence of different compounds for 7 days. ALP^+^ OB differentiation was evaluated after ALP staining.

### Western blot analysis

Cultured BM macrophages by M-CSF from C57Bl6 mice (19, 34) and C3H10T1/2 mouse MSCs, treated with different compounds, were lysed with M-Per mammalian protein extraction reagent (Thermo Scientific) containing a protease inhibitor cocktail (Sigma). Lysates (10–20 μg) were loaded in 10% SDS-PAGE gels and transferred onto polyvinylidene difluoride membranes. Following blocking in 5% milk, membranes were incubated overnight at 4 °C with anti-mouse p100/p52, RelB, RelA, p-IκBα, or β-actin antibody (Ab). After washing, the membranes were incubated with horseradish peroxidase-linked secondary Ab (Bio-Rad). The membranes were exposed to ECL substrate, and signals were analyzed using a Bio-Rad imaging system, which is also used to quantify the band volumes of Western blot.

### NIK kinase activity assay

The inhibition of the compounds on NIK activity was tested using ADP-Glo Kinase Assay kit (Promega, Cat# V4077) following the manual instruction in 96-well plate. The reaction condition was set at NIK kinase protein 5 ng, ATP 50 μM, myelin basic protein substrate 0.1 μg/μl in a volume of 25 μl for 60 min.

### Overexpression of NF-κB subunits

BdMPCs from WT mice in 12-well plates, 1 × 10^4^/well were cultured overnight at 37 °C with 5% CO_2_ followed by treatment with 25% volume of pMX-GFP, pMX-p52, or pMX-p100 retroviral supernatants generated by Plat-E packaging cells and 2 μg/ml polybrene, as we reported (34, 51). Twenty-four hours later, the cells were begun to treat with vehicle (Veh) or 3 μM of plumbagin for 7 days. The cells were fixed with 10% neutral buffered formalin followed by ALP staining to measure the area (Ar.) of ALP^+^ cells.

### Quantitative real time PCR

Total RNA was extracted by Trizol reagent (Invitrogen). Total RNA (1 μg) was reversely transcribed to cDNA in a 20 μl reaction using an iSCRIPT cDNA Synthesis kit (Bio-Rad). The expression levels of *p100/p52* (19) and OB marker genes, Colα1, ALP, osteocalcin, SP7, and Runx2, normalized by β-actin, were measured using an iCycler real time PCR machine (Bio-Rad) with iQ SYBR SuperMix (Bio-Rad) according to the manufacturer’s instruction.

### ELISA

Level of osteocalcin, a specific biomarker of bone formation (79), in serum samples, was tested by ELISA, according to manufacturer's instructions (MyBiosource).

### Statistics

All data were performed normality test. Data were given as the mean ± SD when they were distributed normally. Median and interquartile range were used instead when data distributions were skewed. Comparisons between two groups were analyzed using Student's two-tailed unpaired *t* test and those among three or more groups using one-way analysis of variance followed by Dunnett’s post hoc multiple comparisons when data were distributed normally. In contrast, log-transformed data were used to do statistical analysis when data distributions were skewed. The sample size for *in vivo* experiments is based on an unpaired *t* test power analysis carried out using SigmaStat Statistical Software: seven mice were needed in each group where bone parameters are being assessed to detect significant differences from controls with an alpha error of 5%. The power is 0.98, *i.e.*, there is 98% chance of detecting a specific effect with 95% confidence when alpha=0.05.

## Data availability

All data are available in the main text or the supplementary materials.

## Conflict of interest

The authors declare that they have no conflicts of interest with the contents of this article.
